# Maternal near-miss prediction model development in Bahir Dar city administration, Northwest Ethiopia

**DOI:** 10.1371/journal.pone.0328069

**Published:** 2025-07-10

**Authors:** Yinager Workineh, Getu Degu Alene, Gedefaw Abeje Fekadu

**Affiliations:** 1 Department of Midwifery, College of Medicine and Health Sciences, Bahir Dar University, Bahir Dar, Ethiopia; 2 Department of Epidemiology and Biostatistics, College of Medicine and Health Sciences, Bahir Dar University, Bahir Dar, Ethiopia; 3 Department of Reproductive Health and Population Studies, College of Medicine and Health Sciences, Bahir Dar University, Bahir Dar, Ethiopia; Arba Minch University, ETHIOPIA

## Abstract

**Background:**

Maternal near-miss is a serious public health concern in impoverished countries such as Ethiopia. Despite its huge burden, the prognostic predictive model of maternal near-miss has received little attention in research in the Ethiopian context. As a result, this study aimed to build and validate (internally) a clinical prediction model of maternal near-miss in Bahir Dar City, Northwest Ethiopia, in 2024.

**Methods:**

A prospective follow-up study was conducted among 2110 randomly selected pregnant women in Bahir Dar city between May 1, 2023, and March 6, 2024. Pregnant women with gestational age less than 20 weeks were included in the cohort and followed up to 42 days after delivery. Data were extracted from antenatal care records and collected by an interview-administered questionnaire. The model was developed using the standard Cox regression model, and model fitness was checked using the Schoenfeld assumption test. After applying a stepwise elimination, a p-value of less than 0.15 was used to fit the reduced model. Both discrimination and calibration were used to assess the model’s performance. The model was internally validated through the bootstrapping method. The clinical usefulness of the model was checked using decision curve analysis. A nomogram was used for the model presentation.

**Results:**

Maternal near-miss incidence density rate was 1.94 per 1,000 woman-weeks. Maternal age, residence, decision-making power, intention to pregnancy, time of antenatal initiation, genital mutilation, history of cesarean section, middle upper arm circumference, systolic blood pressure, hemoglobin, and history of obstetric morbidity were identified as important predictors to predict maternal near-miss. The model demonstrated good discriminatory performance with a C-index of 0.82(95%CI: 0.80–0.85), and good calibration with close alignment with 45 degrees. A simplified risk score of 40 maximum points was developed. The model was presented using a nomogram.

**Conclusion:**

The maternal near-miss incidence density rate was high in the present study. Socio-demographic and clinical factors were key variables for predicting maternal near-miss. The model has good discrimination and calibration. The researchers recommend external validation in different settings to assess the model’s generalizability before applying it to clinical settings.

## Introduction

A maternal near-miss is a condition in which a woman faces and escapes a potentially fatal obstetric incident during pregnancy, childbirth, or within 42 days after giving birth [1]. Maternal near-miss enables targeted interventions to address the factors that contributed to women’s actual deaths or near-death experiences [2]. Inadequate maternal healthcare systems can be recognized more quickly using maternal near-miss statistics than maternal mortality [1]. The World Health Organization (WHO) developed maternal near-miss diagnostic tools, including clinical, laboratory, and management-based criteria [3]. These tools were also modified for Sub-Saharan Africa (SSA) [4].

For every maternal mortality, there are roughly 20–30 near-miss incidents, demonstrating a huge gap in the quality of care for pregnant women [5]. The overall burden of near-miss in 2022 was 18.67/1000, with geographical variances ranging from 3.10/1000 in Europe to 31.88/1000 live births in Africa [6]. The maternal near-miss rate in SSA was 24.2 with an interquartile range (IQR) of 12.4 to 35.8 per 1000 live births [7]. The maternal near-miss rate in Ethiopia was 12.57%, with the largest magnitude (26.5%) in the Amhara region [8].

The key risk factors of maternal health include delays in seeking care, reaching healthcare facilities, and receiving adequate treatment [9]. Delays in seeking care often arise from not recognizing complications, underestimating illness severity, cost concerns, negative healthcare experiences, and needing family permission [9,10]. Access to healthcare can be hindered by long distances to facilities, poor road conditions, and limited transportation options [11]. Moreover, delays in obtaining proper care can result from unprofessional provider behavior, lack of supplies, insufficient staff, and a failure to act urgently in emergencies [9].

Different initiatives were developed to enhance maternal health outcomes [12–17]. Clinical prediction models are also essential to support maternal healthcare systems alongside different clinical management. These models use mathematical methods such as parametric, semi-parametric, or non-parametric to estimate the likelihood of diseases or future outcomes [18]. They derive unknowns from known data to calculate probabilities based on known variables [19]. The models help researchers, patients, and healthcare providers improve maternal outcomes through better subject selection, decision-making power, and resource allocation [20]. The clinical prediction models also support primary, secondary, and tertiary disease prevention [21].

Researchers have developed and validated clinical prediction models for severe maternal outcomes to enhance shared decision-making, manage patient expectations, and categorize patients [22–27]. However, the researchers in Ethiopia did not thoroughly investigate the risk categorization or stratification of obstetric patients based on prognostic predictors using a prospective follow-up approach. The lack of prognostic prediction models for maternal near-miss in Ethiopia indicates the need to develop and validate models based on easily identifiable demographic and clinical factors. This model will identify high-risk women and help prevent maternal near-miss. Therefore, this study aimed to determine incidence density and develop a validated prognostic prediction model and risk score for maternal near-miss events.

## Methods

### Study design and period

A prospective follow-up study was conducted to develop a prognostic prediction model of maternal near-miss. The theoretical design of the study suggests that the incidence of maternal near-miss at a future time “t” is influenced by various prognostic factors measured earlier at “t0,” during pregnancy or after delivery up to 42 days. The domain was pregnant women who started antenatal care (ANC) within 20 weeks of gestational age (GA). Maternal near-miss prognostic index (PI) =f (D1, D2, D3 …Dn).

Where:

PI = Prognostic Index

D1…Dn = Prognostic predictors

Individual-level variables were used as prognostic predictors (Dn) to forecast the incidence of maternal near-miss during the follow-up period. The occurrence of maternal near-miss among pregnant women as a function of individual-level predictors is expressed as=f (age, residence, decision-making power…Dn). The probability of experiencing the event can be calculated as:


$$1-S(t)\;=1-S_{o}{\left(t\right)}^{exp(PI)}\mathrm{\backslash ]}$$

Where:

S(t) = Survival function at time t.

S_o_(t) = Baseline survival function at time t.

The study was conducted from May 1, 2023 to March 6, 2024. The recruitment of participants and data collection on prognostic predictors were performed from May 1, 2023 to September 1, 2023 (baseline period). The follow-up assessment of the participants was from May 2, 2023 to March 6, 2024 (end-line period). The follow-up assessment was carried out at any time in women who encountered a problem, whereas in a normal situation, the assessment of the participants was conducted in line with the maternal routine care timeline. Hence, the timeline of follow-up assessment was at 26, 30, 34, 36, 38, and 40 weeks of GA, during childbirth, and at 42 days after delivery.

#### Study setting and participants’ recruitment.

The study was carried out in the Bahir Dar City administration in Northwest Ethiopia. Bahir Dar is 484 km away from Addis Ababa. This city has both urban and rural populations. It has three public hospitals, eleven healthcare centers, fifteen health posts, four private hospitals, fifty-six private specialty clinics, and thirteen private medium clinics [28].

**Fig 1:**
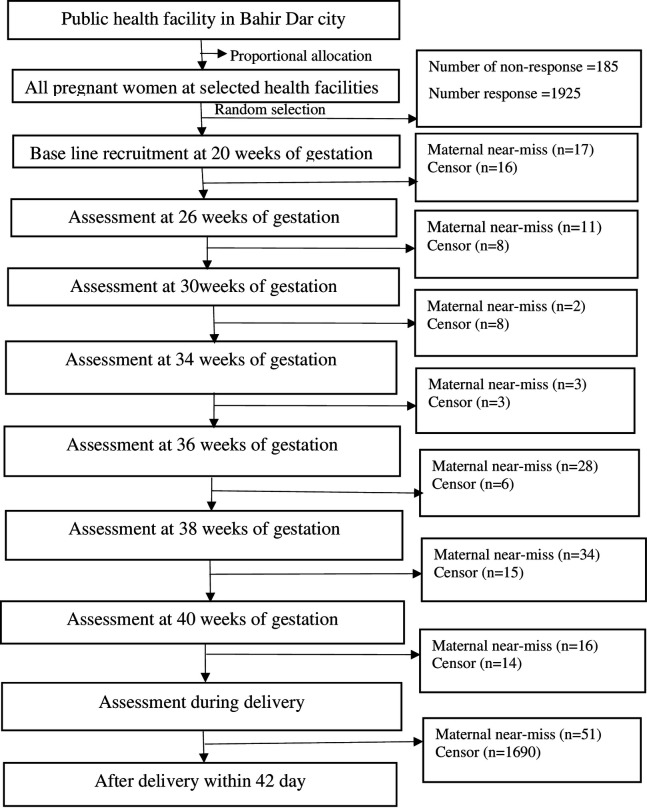
Flow chart for participants’ recruitment in Bahir Dar city, northwest Ethiopia, 2024.

This study included public health facilities in the Bahir Dar City administration that provide ANC services. Four health facilities with senior physicians were included. The total sample size was proportionally allocated to each health facility based on last year’s reports. Then, pregnant women who came for their ANC visit within 20 weeks of GA were selected using a systematic sampling method. In this regard, the woman who arrived first in the ANC clinic was chosen as a starting participant on each data collection day. Then, every other visitor was selected. Lastly, the selected pregnant women were followed until they either experienced the event or censorship. The event group consisted of women who experienced maternal near-miss. Only the initial occurrence was included if a woman had several maternal near-miss. The censoring group included women who withdrew from ANC visits, were referred out from the selected healthcare facility, changed the selected health facility, or the women completed the follow-up period without event. During the baseline period, 185 women refused to participate. Among the 1,925 participants, 165 experienced maternal near-miss, 1,702 completed the cohort without an event, 22 changed health facilities, 34 withdrew from participation, and 4 were referred to other facilities (Fig 1).

### Eligibility criteria

Women who had no plan to relocate from the study area and were considered well enough to be interviewed by the interviewer were included in the study. Pregnant women who did not remember their last normal menstrual period were excluded from the study.

### Outcome measurement

Maternal near-miss event was diagnosed during the follow-up phase using the WHO disease-based screening criteria [4]. This screening criteria includes acute cyanosis (0 = no, 1 = yes), gasping (0 = no, 1 = yes), respiratory rate > 40 or < 6 breaths/minute (0 = no, 1 = yes), shock (0 = no, 1 = yes), oliguria non-responsive to fluids or diuretics (0 = no, 1 = yes), failure to form clots (0 = no, 1 = yes), loss of consciousness lasting 12 hours (0 = no, 1 = yes), cardiac arrest (0 = no, 1 = yes), stroke (0 = no, 1 = yes), uncontrollable fit/total paralysis (0 = no, 1 = yes), jaundice in the presence of pre-eclampsia (0 = no, 1 = yes), eclampsia (0 = no, 1 = yes), uterine rupture (0 = no, 1 = yes), pulmonary edema (0 = no, 1 = yes), severe malaria (0 = no, 1 = yes), severe pre-eclampsia with intensive care unit admission (0 = no, 1 = yes), sepsis or severe systemic infection (0 = no, 1 = yes), or severe abortion complications (0 = no, 1 = yes). Women who met at least one of these criteria were classified as the event group, while the remaining were classified as the censored group. The censored group comprised women who changed their ANC facility to outside the study site, referred to areas beyond the study site, withdrew from participation, or the women completed the follow-up period without event. Survival time was calculated from the last normal menstrual period to the event or censoring. Hence, the survival time was measured in weeks (s) from the previous date of the normal menstrual period to the occurrence of maternal near-miss or censorship.

### Predictors and measurements

Individual-level data on prognostic predictors were gathered using an interview-administered questionnaire, measurements, and an extraction sheet. The socio-demographic variables included age (measured in a year), maternal educational status (‘1’ no formal education, ‘2’ primary & secondary, and ‘3’ above secondary), maternal occupational status (‘1’ for housewife, ‘2’ employed, ‘3’ for merchant and ‘4’ for farmer), marital status (coded as ‘1’ for married, ‘2’ for single, ‘3’ for widowed and ‘4’ for divorced), residence (coded as ‘1’ for rural and ‘0’ for urban), decision-making for healthcare (coded as ‘1’ for husband or third party, and ‘0’ for self or jointly), and mid-upper arm circumference (MUAC) (measured in centimeters). Additionally, obstetric variables included gravidity (measured in number), parity (measured in number), plurality (coded as ‘0’ for single and ‘1’ for multiple), pregnancy intention (coded as ‘1’ for unintended and ‘0’ for intended), inter-pregnancy interval (IPI) (measured in month), and history of cesarean section (C/S) (coded as ‘1’ for yes and ‘0’ for no). Other factors considered were history of obstetric morbidity (coded as ‘1’ for yes and ‘0’ for no), history of low birth weight (coded as ‘1’ for yes and ‘0’ for no), history of medical morbidity (coded as ‘1’ for yes and ‘0’ for no), history of stillbirth (coded as ‘1’ for yes and ‘0’ for no), history of abortion (coded as ‘1’ for yes and ‘0’ for no), distance from the health facility (measured in kilometer), timing of initial antenatal contact (measured in weeks), systolic blood pressure (measured in mmHg), diastolic blood pressure (measured in mmHg), hematocrit value (percentage), and hemoglobin value (mg/dL).

### Blinding

Blinding reduces the risk of bias introduced in the model development. This risk of bias was solved by measuring the predictors at the baseline and the outcome at the follow-up period by different data collectors.

### Sample size determination

The sample size was determined based on the sample size calculation criteria for a time-to-event study. The minimum criteria for this calculation are: 1) a minimum heuristic shrinkage factor, S, greater than 0.9 (targeting less than 10% over-fitting), 2) a small difference between Nagelkerke’s R2app and R2adj (targeting less than 0.05 absolute differences), and 3) a small margin of error in the overall risk estimate (targeting less than 0.05 absolute errors). These criteria, including the number of parameters (P), heuristic shrinkage factor (S), the overall risk in the population, and the model’s anticipated Cox-Snell R2 (or C-statistics) were reviewed from previous studies. Approximately 25 candidate parameters, 26.6% of maternal near-miss [29], and C-statistics of 0.11 [27] were utilized to calculate the sample size. The sample size was then calculated using the Stata command “pmsampsize, type (b) rsquared (0.11) parameter (25) prev (0.266)”. The resulting sample size was 1918 (Table 1).

**Table 1:** Sample size calculation for prognostic prediction model development of maternal near-miss using minimum criteria of event-to-time method in Bahir Dar city, Ethiopia 2024.

Criteria	Sample size	Shrinkage	Parameter	Rsq	Max-Rsq	EPP
**Criteria1**	1918	0.90	25	0.11	0.52	9.21
**Criteria** 2	895	0.81	25	0.11	0.52	4.30
**Criteria** 3	162	0.90	25	0.11	0.52	0.78
**Final**	1918	0.90	25	0.11	0.52	9.21

Finally, considering a 10% non-response rate, the sample size for developing a predictive model for maternal near-miss was 2110.

#### Data collection process.

Baseline data and follow-up data were collected by eight (four in each period) data collectors (Bachelor’s degree in Midwifery). A supervisor (Epidemiologist) supervised the data collection process. Data-gathering tools for this study included direct participant measurements, exit interview questions for women at ANC, and checklists to extract clinical outcomes from maternal cards and ANC charts.

During the baseline data collection process, data collectors identified women within 20 weeks of GA at the ANC clinic. They directly measured key variables using tools, including the last normal menstrual period, expected date of delivery, MUAC, and systolic and diastolic blood pressure. Additionally, obstetric history, medical history, and laboratory findings were abstracted from the women’s ANC charts and cards using checklists. Finally, each pregnant woman was interviewed on socio-demographic characteristics, wealth index, and obstetric-related factors at the ANC exit every day.

During the follow-up data collection period, aligned with the routine timeline of ANC visits, data collectors extracted information on obstetric complications, including maternal death and maternal near-miss cases, as well as reasons for censorship and their timing from the ANC charts and maternal cards. Mid-upper arm circumference, systolic, and diastolic blood pressure were taken from participants using measurement tools. At the time of delivery, data on obstetric complications, including maternal near-miss and maternal death, along with birth outcomes and their timing, were collected from delivery charts and maternal cards using extraction checklists. Additionally, within 42 days post-delivery, data on postnatal complications, such as maternal death and maternal near-miss cases, as well as reasons for censorship and their timing, were gathered from the maternal card and postnatal charts using extraction checklists (S1 File).

### Data quality assurance

Experts evaluated the questionnaire for face and content validity. Data collectors and supervisors received two days of training to become familiar with the questionnaires, data collection processes, ethical considerations, and the purpose of the study. Similarly, health professionals who work in antenatal, delivery, and postnatal departments were informed of the screening criteria for maternal near-miss. Then, a pretest was conducted by data collectors and a supervisor. This pretest was undertaken to ensure the accuracy of the data and to check for ambiguities in language after translating from English to Amharic. The pretest enabled us to modify the question order and refine our data collection instruments. It assisted us in selecting the appropriate tools to measure the variables under investigation. The pretest also determined how much time would be required for data collection before the actual data collection time. The supervisor performed daily supervision and verification of all collected data. The data was regularly checked for completeness, and any problems during collection were addressed appropriately. Finally, the Transparent Reporting of a Multivariable Prediction Model for Individual Prognosis or Diagnosis (TRIPOD) guideline was utilized to develop and report the prediction model [18].

### Data processing and management

The baseline data were collected using Epi-Collect5, while the follow-up data were collected using a hard copy questionnaire. The data from Epi-Collect5 was downloaded and transferred to Microsoft Excel. The illogical values and steps were checked during the questionnaire design in Epi-Collect5. The collected data was examined for consistency and completeness. The baseline data was transferred to SPSS version 23.0, and the follow-up data entry was conducted. Then the data was exported to R 4.2.2 software for analysis.

Once the data was prepared for analysis, a thorough evaluation was conducted to identify missing data. Missing data is a common problem that can impact the accuracy of classification and the models generated from data. In the present study, complete case analysis and imputation were applied. In the case of predictor variables such as history of multiple pregnancies, IPI, and history of C/S that had missing values due to skipping patterns during the questionnaire design, exclusion cases with missing values were applied. Additionally, missing values were replaced with randomly generated values from the observed variable distribution. In this regard, multiple imputation techniques were used when the amount of missing data was less than 10%. The steps of multiple imputations involve: 1) replacing missing values with randomly selected values from specific distributions to create complete case datasets, 2) conducting the same analysis on each of these datasets, and 3) pooling the results together. This imputation process was repeated 5 times. In this study, hematocrit and hemoglobin values were imputed.

After handling the missing data, data transformations, such as normalization, were applied to remove noise and correct inconsistencies in the data. For continuous variables, dichotomization or categorization was performed based on widely accepted clinical cutoff values.

The distribution of continuous variables was examined graphically using a histogram and statistically using the Shapiro-Wilk test. If the data followed a normal distribution, the mean, with standard deviation (SD), was used to present the data, whereas if the data did not follow a normal distribution, the median, with an interquartile range (IQR), was used, and data transformation and standardization were performed. Multicollinearity between each independent predictor was checked using the variance inflation factor (VIF). If the VIF is less than 10, there is no multicollinearity. In this regard, diastolic blood pressure, parity, and hematocrit value were excluded due to their strong collinearity with systolic blood pressure, gravidity, and hemoglobin value, respectively.

### Statistical analysis methods

#### Predictor selection.

The prognostic predictors were chosen based on previously identified predictors, the feasibility and costs associated with measuring these predictors in the specific context, clinical relevance, and the statistical power of the analysis.

#### Model estimation and specification.

Both bivariable and multivariable Cox proportional hazard regression models were utilized, with the strength of the association measured as absolute risk (β coefficient) at a 95% confidence interval. The assumptions of this model were checked using the scaled Schoenfeld residual test. Variables with a p-value of < 0.25 from the bivariable analysis were included in the multivariable regression analysis. After applying a stepwise elimination, each predictor’s contribution was assessed using the likelihood ratio test; a p-value less than 0.15 was used for fitting the reduced model.

#### Model performance.

Model predictions can be assessed over the entire range of observed follow-up times or for events occurring by a specified time horizon of interest. Discrimination, calibration, and overall performance are essential to evaluate the model’s accuracy [30].

**Discrimination:** The initial question concerns the model’s ability to distinguish between high-risk and low-risk patients, known as its discriminative ability. Patients who experience an event sooner should demonstrate a higher risk, while those with a later event should show a lower risk. The discrimination ability of the prognostic clinical model was assessed based on time-range discrimination [31]. The values ranging from 0.9 to 1.0, 0.8 to 0.9, 0.7 to 0.8, 0.6 to 0.7, and 0.5 to 0.6 were classified as excellent (A), good (B), fair (C), poor (D), and fail (F), respectively. Harrell’s concordance index (C) is commonly used for time-range discrimination to assess overall performance [32].

**Calibration:** Calibration evaluates whether the predicted hazards align with the actual hazards [33]. It can be assessed using a calibration plot. Calibration can also be determined based on time range calibration. Mean calibration (calibration-in-the-large) refers to the alignment between predicted and observed survival fractions. Fixed time point mean calibration is usually expressed as the ratio of the observed survival fraction to the average predicted risk. Mean calibration can be assessed by comparing observed and predicted event counts for time range calibration [31].

#### Internal validation.

Different techniques exist for internal verification of prognostic forecasting models, including cross-validation, bootstrapping, and split-sample validation [34]. The bootstrapping approach surpasses the split-sample and cross-validation methods in addressing optimism. Therefore, this study utilized 10,000 random bootstrap samples with replacement on all predictors in the dataset to validate the model.

#### Overall performance.

The Brier score is a widely used metric for overall performance up to a fixed time point that includes both discrimination and calibration [35]. It incorporates inverse weights and is calculated as the mean squared difference between observed survival and predicted risk. The Brier score of 0 indicates a perfect model. In this study, an integrated Bier score was used to address complicating interpretation [36].

Decision curve analysis (DCA) is a commonly employed method to evaluate the efficacy of clinical prediction models. Traditional measures of diagnostic performance, such as sensitivity, specificity, and area under the receiver operating characteristic curve, do not consider the clinical value of a specific model. Instead, these measures only compare the diagnostic accuracy of different prediction models [37]. Therefore, DCA was utilized to assess the clinical and public health impacts of the maternal near-miss model.

#### Development of a simplified risk score.

After developing the prediction model, a simplified risk score was produced. Predictor coefficients were converted to whole numbers and rounded, with total scores calculated by assigning points for each variable and summing up them. Continuous predictor variables were categorized based on clinical guidelines. The reference class was set by the direction of the beta coefficients using the ‘last class’ for negative and the ‘first class’ for positive. A multiplication factor was calculated by subtracting the reference class mean from each class mean. Each class’s beta coefficient was obtained by multiplying the continuous variable’s beta by this factor and dividing by the smallest coefficient to assign scores.

#### Model presentation.

Once the model was developed and validated, a risk score was generated for maternal near-miss. This score was based on predictors at the individual level and will be user-friendly. The coefficients of each predictor that are statistically significant in the multivariable Cox proportional hazard regression model were adjusted to calculate the risk scores. The risk score and the nomogram development cut-off point were determined using the Youden index value (sensitivity + specificity – 1) for each risk category. Finally, a simplified nomogram was developed for potential users as part of the clinical prediction model.

**Fig 2:**
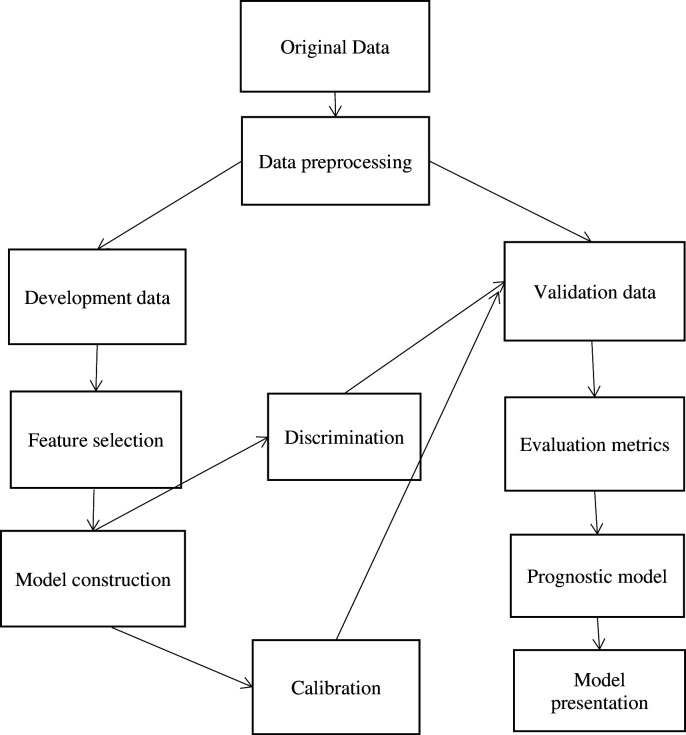
Computational workflow of the study to develop a maternal near-miss prediction model in Bahir Dar city, Northwest Ethiopia, 2024.

The following figure represents the overall computational workflow of the study (from data preprocessing to the construction of a prognostic model) (Fig 2).

### Ethical statement

The Institutional Review Board of the College of Medicine and Health Sciences, Bahir Dar University, has granted ethical clearance (protocol number 704/2023) for this study. The participating mothers were provided with information about the purpose, benefits, and their right to decline participation in the research. The interviewers thoroughly explained the contents of the information sheet and consent form to ensure understanding. Participants indicated their agreement or disagreement through written consent, with signatures for the educated and fingerprints for the non-educated. To uphold anonymity and confidentiality, each participant was assigned a unique code.

## Results

A total of 2,110 pregnant women were selected as study participants. During the baseline recruitment, 1,925 women participated, yielding a response rate of 91.2%. From the 1925 participants, 165 events, and 1760 censors occurred during the follow-up period. The majority of maternal near-miss occurred during the antepartum period, 98(59.39%), followed by the postpartum period, 51 (30.91%), and the intrapartum period, 16(9.70%).

The maternal near-miss incidence density rate was determined using person-weeks as the denominator for the entire cohort, resulting in 85,072 woman-weeks of observation. Follow-up times ranged from 21 to 47.43 weeks. The maternal near-miss overall incidence density rate was 1.94 per 1,000 woman-weeks. Throughout the follow-up period, maternal near-miss cases varied, with high rates from 38–40 weeks and low rates from 28–32 weeks of follow-up (Table 2).

**Table 2:** Life table showing the maternal near-miss in a public health facility, Bahir Dar City Northwest Ethiopia, 2024.

Interval in week		Women at risk	Maternal near-miss	Censored	Survived	SD.error	95% CI	
							LB	UB
20	22	1925	0	2	1.0000	0.0000	..	
22	24	1923	3	5	0.9984	0.0009	0.9952	0.9995
24	26	1915	14	9	0.9911	0.0021	0.9858	0.9945
26	28	1892	9	3	0.9864	0.0026	0.9801	0.9907
28	30	1880	2	5	0.9854	0.0027	0.9789	0.9899
30	32	1873	2	5	0.9843	0.0028	0.9776	0.9890
32	34	1866	0	3	0.9843	0.0028	0.9776	0.9890
34	36	1863	3	3	0.9827	0.0030	0.9758	0.9877
36	38	1857	43	6	0.9599	0.0045	0.9501	0.9679
38	40	1808	66	15	0.9247	0.0061	0.9119	0.9358
40	42	1727	18	14	0.9151	0.0064	0.9015	0.9268
42	44	1695	2	31	0.9140	0.0065	0.9004	0.9258
44	46	1662	3	1274	0.9113	0.0066	0.8974	0.9234
46	48	385	0	385	0.9113	0.0066	0.8974	0.9234

### Demographic characteristics

The event group (46.58%) was more likely to be above 35 years old than the censored group (16.03%). The event group was more likely to live in a rural area (34.16%) than the censored group (9.58%). The event group (23.60%) was more likely to make decisions by her husband or a third party than the censored group (6.85%). Similarly, the event group (49.07%) was more likely to live more than four km from health than censored group (13.44%) (Table 3).

**Table 3:** Demographic characteristics of participants in a public health facility, Bahir Dar City, Northwest Ethiopia, 2024.

Prognostic factors		Event	Censored
		n(%)	n(%)
**Age**	18 −24 years	12(7.45)	145(9.65)
	25 −34 years	74(45.96)	1117(74.32)
	35 + year	75(46.58)	241(16.03)
**Religion**	Orthodox	144(89.44)	1346(89.55)
	Muslim	11(6.83)	117(7.78)
	Protestant	6(3.73)	40(2.66)
**Ethnicity**	Amhara	157(97.52)	1460(97.14)
	Others^*^	4(2.48)	43(2.86)
**Maternal educational status**	No formal education	23(14.29)	197(13.11)
	Primary	59(36.65)	572(38.06)
	Secondary	60(37.27)	618(41.12)
	Above secondary	19(11.80)	118(7.85)
**Maternal occupational status**	Housewife	87(54.04)	813(54.09)
	Employed	41(25.47)	437(19.08)
	Merchant	17(10.56)	123(8.18)
	Farmer	16(9.94)	130(0.65)
**Marital status**	Married	153(95.03)	1442(95.94)
	Single	6(3.73)	45(3.00)
	Widowed	2(1.24)	16(1.06)
**Residence**	Urban	106(65.84)	1359(90.42)
	Rural	55(34.16)	144(9.58)
**Decision**-**making power**	Self/jointly	123(76.40)	1400(93.15)
	Husband/third party	38(23.60)	103(6.85)
**Wealth index**	Poorest	24(14.91)	284(18.90)
	Poor	36(22.36)	294(19.56)
	Medium	38(23.60)	302(20.09)
	Rich	26(16.15)	315(20.96)
	Richest	37(22.98)	308(20.49)
**Distance to a health facility in km**	1-2 km	67(41.61)	580(38.59)
	3-4 km	15(9.32)	721(47.97)
	Greater than 4 km	79(49.07)	202(13.44)

* Tigray, Guragai, and Oromo.

### Clinical factors

Unintended pregnancies were more prevalent in the event group (44.10%) than in the censored group (29.54%). Female genital mutilation was also more common in the case group (32.92%) compared to the censored group (7.85%). A history of C/S was more frequent in the event group (31.06%) than in the censored group (6.92%). Additionally, a history of obstetric morbidity was more common in the event group (14.91%) compared to the censored group (7.92%). Having MUAC less than 23 cm was more common in the event group (27.74%) than the censored group (9.98%). Having ≥ 140 mm Hg of systolic blood pressure was more common in the event group (18.01%) than the censored group (5.06%). Similarly, having a hemoglobin level of 7 g/dl to 8.9g/dl was more common in the event group (3.12%) than the censored group (0.26%) (Table 4).

**Table 4:** Clinical-related factors of participants in a public health facility, Bahir Dar City, Northwest Ethiopia, 2024.

Prognostic factors		Event	Censored
		n (%)	n (%)
**Interpregnancy interval**	Less than 18 months	5(3.11)	94(6.25)
	18 −24 months	86(53.42)	480(31.94)
	More than 24 months	70(43.48)	929(61.81)
**Time of antenatal care initiation**	Less than 12 weeks of GA	47(29.19)	641(42.65)
	13-16 weeks of GA	23(14.29)	563(37.46)
	17 + weeks of GA	91(56.52)	299(19.89)
**Intention of pregnancy**	Intended	90(55.90)	1059(70.46)
	Unintended	71(44.10)	444(29.54)
**Gravidity**	Multigravida	154(95.65)	1412(93.95)
	Grand multigravida	7(4.35)	91(6.05)
**Parity**	Primi parous	46(28.57)	424(28.21)
	Multiparous	114(70.81)	1074(71.46)
	Grand multiparous	1(0.62)	5(0.33)
**History of plurality**	Single	154 (95.65)	1,456 (96.74)
	Multiple	7(4.35)	49(3.26)
**History of abortion**	No	154 (95.65)	1,454 (96.74)
	Yes	7(4.35)	49(3.26)
**History of low birth weight**	No	159 (98.76)	1,474(98.07)
	Yes	2 (1.24)	29 (1.93)
**History of stillbirth**	No	160 (99.38)	1,475(98.14)
	Yes	1 (0.62)	28 (1.86)
**Female genital mutilation**	No	108(67.08)	1385(92.15)
	Yes	53(32.92)	118(7.85)
**History of C/S**	No	111(68.94)	1,399(93.08)
	Yes	50(31.06)	104(6.92)
**History of obstetric morbidity**	No	137(85.09)	1384(92.08)
	Yes	24(14.91)	119(7.92)
**Middle upper circumference**	Less than 2 3 cm	35(27.74)	150(9.98)
	23 cm or greater	126(78.26)	1353(90.02)
**Systolic blood pressure**	< 120 mm Hg	3(1.86)	43(2.86)
	120–129 mm Hg	75(46.58)	846(56.29)
	130–139 mm Hg	54(33.54)	538(35.78)
	≥ 140 mm Hg	29(18.01)	76(5.06)
**Diastolic blood pressure**	< 80 mm Hg	24(14.91)	559(37.19)
	80–89 mm Hg	107(66.46)	876(58.28)
	≥ 90 mm Hg	30(18.63)	68(45.24)
**Hemoglobin value**	7-8.9 g/dl	5(3.12)	4(0.26)
	9-10.9 g/dl	37(22.98)	51(3.39)
	≥11 g/dl	119(73.91)	1448(96.34)
**History of medical morbidity**	No	140 (86.96)	1391(92.55)
	Yes	21 (13.04)	112(7.45)

### Prognostic predictor selection

The selection of prognostic predictors was based on statistical power and clinical relevance of the predictors. We used Cox proportional hazard regression to construct a maternal near-miss model. The proportionality assumption for each predictor variable was assessed and satisfied (global p-value = 0.783). Bivariable Cox proportional hazards regression analysis indicated that maternal age, residence, wealth index, decision-making power, distance, pregnancy intention, MUAC, the timing of ANC initiation, gravidity, female genital mutilation, history of C/S, IPI, systolic blood pressure, hemoglobin level, history of medical and obstetric morbidities had a p-value < 0.25. In the multivariable analysis, maternal age, residence, decision-making power, pregnancy intention, timing of ANC initiation, female genital mutilation, history of C/S, systolic blood pressure, hemoglobin level, MUAC, and history of obstetric morbidity were statistically significant at a p-value of < 0.15 (Table 5).

**Table 5:** Cox proportional hazard regression analysis of predictors of maternal near-miss in a public health facility, Bahir Dar city Northwest Ethiopia, 2024.

Prognostic factors	Bivariable model		Full model		Reduce model		Bootstrap model
	B (95%CI)	P value	B (95%CI)	P value	B (95%CI)	P value	B (95%CI)
Age	0.15(0.12, 0.18)	<0.001	0.12(0.08, 0.15)	<0.001	0.12(0.09, 0.15)	<0.001	0.12(0.09, 0.15)
Maternal education- no formal education	−0.31-0.92, 0.30)	0.315	NA		NA		NA
Maternal occupation-farmer	0.15(−0.39, 0.68)	0.582	NA		NA		NA
Residence-Rural	1.47(1.14, 1.79)	<0.001	0.68(0.31, 1.05)	<0.001	0.68(0.33, 1.03)	<0.001	0.68(0.33, 1.03)
Decision-husband/third party	1.30(0.94, 1.66)	<0.001	0.71(0.31, 1.11)	<0.001	0.74(0.28, 1.06)	<0.001	0.74(0.28, 1.06)
Wealth index-poorest	−0.35(−0.85, 0.18)	0.198	−0.01(−0.53, 0.54)	0.969	NA		NA
Pregnancy intension-unintended	0.61(0.30, 0.92)	<0.001	0.45(0.13, 0.78)	0.007	0.47(0.15, 0.79)	0.004	0.47(0.15, 0.79)
Abortion-yes	0.32(−0.44, 1.07)	0.411	NA		NA		NA
Stillbirth-yes	−0.09(−3.06, 0.87)	0.276	NA		NA		NA
Low birth weight-yes	−0.43(−1.82, 0.97)	0.550	NA		NA		NA
Distance	0.18(0.08, 0.28)	<0.001	−0.02(−0.13, 0.08)	0.673	NA		NA
Inter-pregnancy interval	−0.08(−0.12,-0.04)	<0.001	−0.03(−0.07, 0.02)	0.253	NA		NA
Antenatal care initiation time	0.17(0.12, 0.22)	<0.001	0.14(0.09, 0.20)	<0.001	0.15(0.10, 0.20)	<0.001	0.15(0.10, 0.20)
Gravidity in number	0.43(0.26, 0.59)	<0.001	0.08(−0.11, 0.26)	0.420	NA		NA
Gestation-multiple	0.26(−0.50, 1.01)	0.506	NA		NA		NA
Genital mutilation-yes	1.58(1.25, 1.91)	<0.001	0.39(−0.09, 0.87)	0.108	0.42(−0.05, 0.88)	0.081	0.42(−0.05, 0.88)
History of C/S-yes	1.63(1.30, 1.96)	<0.001	0.86(0.39, 1.33)	<0.001	0.86(0.39, 1.32)	<0.001	0.86(0.39, 1.32)
History of obstetric morbidity -yes	0.66(0.23, 1.09)	0.002	0.58(0.13, 1.03)	0.011	0.56(0.12, 1.01)	0.01	0.56(0.12, 1.01)
MUAC in cm	−0.25(−0.33,-0.17)	<0.001	−0.13(−0.24,-0.04)	0.006	−0.13(−0.22,-0.04)	0.004	−0.13(−0.22,-0.04)
Systolic blood pressure	0.07(0.05, 0.09)	<0.001	0.04(0.02, 0.06)	<0.001	0.04(0.02, 0.07)	<0.001	0.04(0.02, 0.07)
Hemoglobin value	−0.56(−0.64,-0.47)	<0.001	−0.47(−0.58,-0.35)	<0.001	−0.48(−0.59,-0.38)	<0.001	−0.48(−0.59,-0.38)
Medical morbidity history-yes	0.58(0.12, 1.04)	0.014	−0.06(−0.56, 0.44)	0.812	NA		NA

### Risk prediction model

Prognostic index of the model is expressed as: PI = 0.12 × age + 0.68 × residence + 0.74 × decision-making power + 0.47 × intention of pregnancy + 0.15 × timing of ANC initiation −0.13 × MUAC + 0.86 × history of C/S + 0.42 × genital mutilation + 0.04 × systolic blood pressure-0.48 × hemoglobin + 0.56 × history obstetric morbidity.

The probability of experiencing the event within 48 weeks of the perinatal period can be calculated as 1-S (t) =1-S_o_ (t)^exp(PI)^ =1-S (48) =1-(0.9106)^exp(PI)^. The survival rate at 48 weeks is 0.9106, which is relevant for the reference categories of the predictors in the model. For instance, a 25 years of age woman from an urban residence, with a health care decision made by herself, having intended pregnancy, no history of genital mutilation, no history of C/S, started ANC at 11 weeks, has a systolic blood pressure of 118 mmHg, MUAC of 24 cm, hemoglobin value of 13 mg/dl and no history of obstetric morbidity, has an estimated risk of maternal near-miss of 1–0.9113, which equals 8.87%, during the 48 weeks of the perinatal period.

### Model performance measurement

Because there were a small number of events in the present study, time-interval discrimination and calibration were used to evaluate model performance. The AUC was calculated using actual survival outcomes and model predictions, with a time interval of 48 weeks. Hence, this method evaluates the model’s overall performance and capabilities.

### Model discrimination

**Fig 3:**
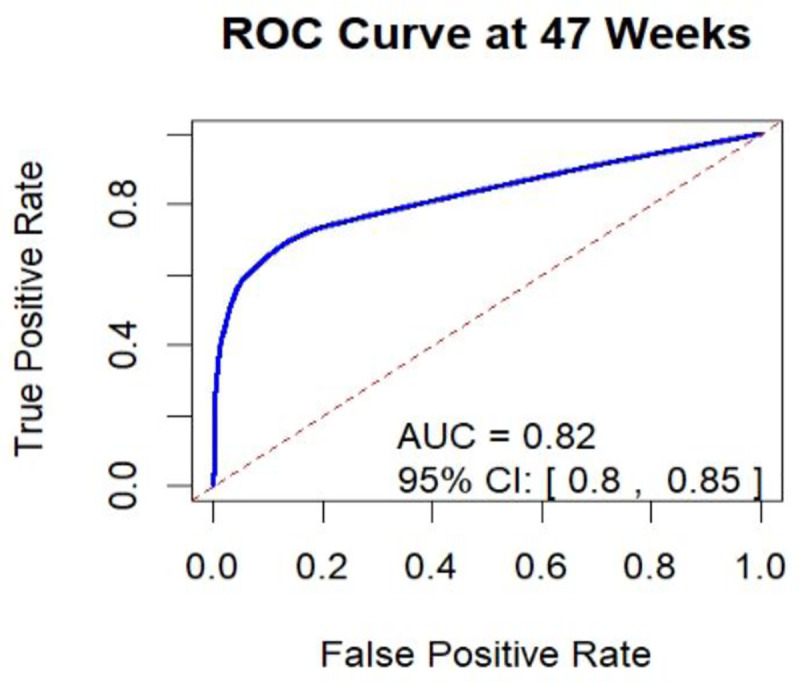
Receiver operating curve with area under the curve of the original model of maternal near-miss in a public health facility, Bahir Dar City Northwest Ethiopia, 2024.

The AUC of the receiver operating characteristics curve of the original model was 0.82(95% CI: 0.80–0.85) (Fig 3).

The prediction model was developed based on the reduced model. This model (based on the default 0.5 cut-off probability) had an accuracy rate of 63.70%, a sensitivity of 20.03%, a specificity of 98.59%, a positive predictive value (PPV) of 91.93%, and a negative predictive value (NPV) of 60.68%. On the other hand, based on the optimal cut-off point (Youden index cut-off point) 1.3662 predicted probability, the model had an accuracy of 83.53%, 95% CI: (81.58, 86.27), sensitivity of 33.99%, specificity of 96.87%, PPV of 74.53%, and NPV of 84.50%.

### Density plot

**Fig 4:**
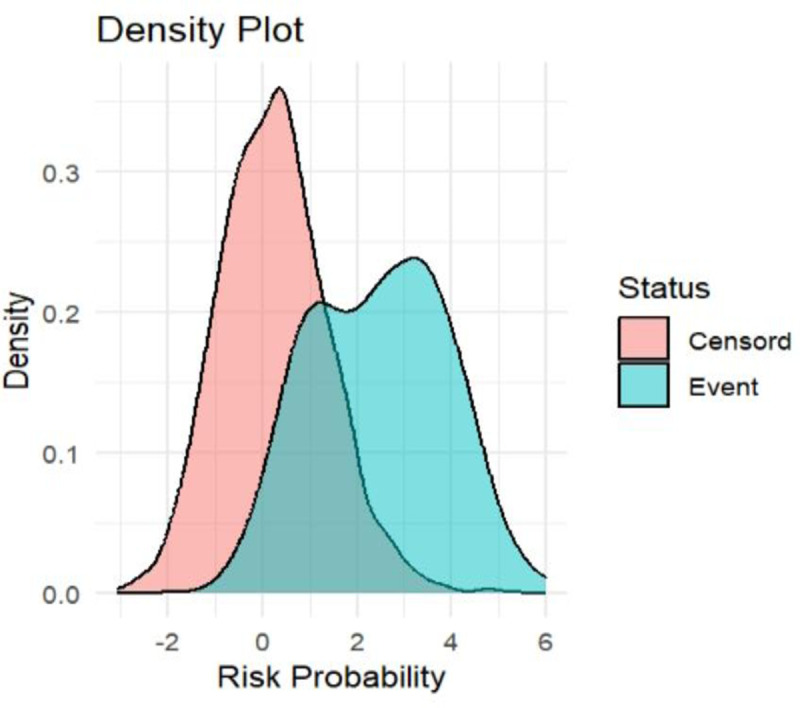
Prediction density of the developed model of maternal near-miss in a public health facility, Bahir Dar City, Northwest Ethiopia, 2024.

The green density plot represented patients with events, while the grey one represented the censored groups. The model’s overlap was plotted, indicating that it isn’t entirely effective at distinguishing between the censord groups and events (Fig 4).

### Model calibration

**Fig 5:**
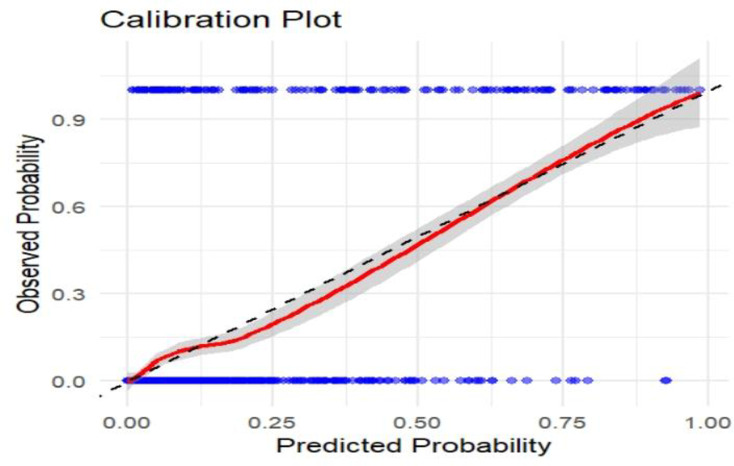
Calibration plot of the original model of maternal near-miss in a public health facility, Bahir Dar City, Northwest Ethiopia, 2024.

The calibration curve has a 45-degree slope, indicating that the predicted value aligns with the observed value (Fig 5).

#### Model validation.

**Fig 6:**
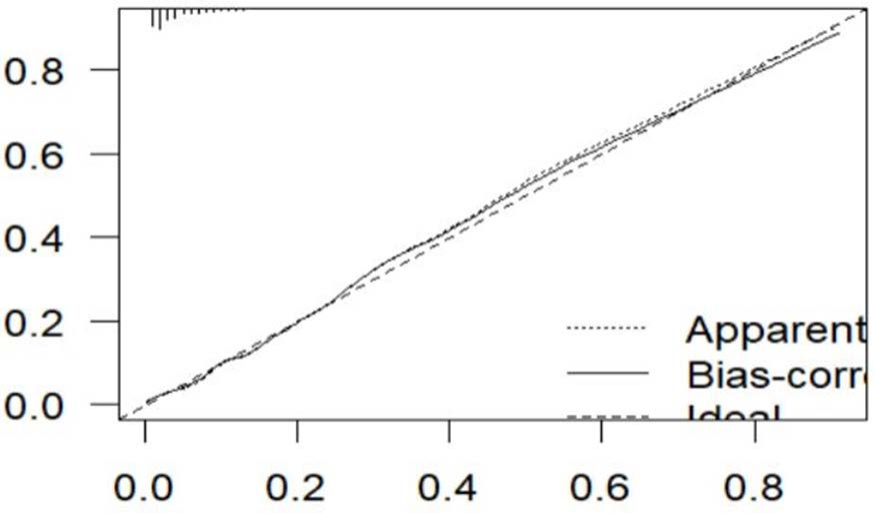
Calibration plots for the validated model of maternal near-miss in a public health facility, Bahir Dar City Northwest Ethiopia, 2024.

A bootstrapping technique was employed to optimize the model and avoid overfitting. After 10000 bootstrap samples with replacement were used, the corrected C-index was 77%, the optimism coefficient for the validated model was 0.06, and the original C-index was 82%. The β coefficients from the bootstrapped model produced the same results as the original β coefficients (Table 5). The calibration plot for the validated model indicated good agreement between the predicted and observed probabilities (Fig 6).

### Overall model performance

**Fig 7:**
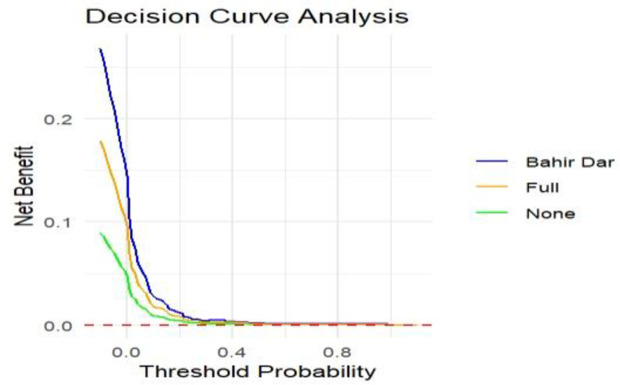
The decision curve of the validated model of maternal near-miss in a public health facility, Bahir Dar City Northwest Ethiopia, 2024.

An integrated Bier score and decision curve analysis measured the overall model performance. The integrated Bier score was 0.016. This value suggests that the model has relatively good predictive performance. This indicates that the predicted probabilities are reasonably close to the observed outcomes. Additionally, we have evaluated the DCA to apply this model to clinical decision-making. The figure below shows that the decision curve outperforms the default strategies (referring to all and none) across the entire range of threshold probabilities. This implies that our model has the highest clinical importance and can facilitate decision-making (Fig 7).

### Development of a simplified risk score

After developing the prediction model of maternal near-miss, a simplified risk score was generated for ease of use. The predictors’ coefficients were converted to whole numbers based on the smallest coefficient and then rounded to the nearest integer. The total score for each individual was calculated by assigning points for each variable present and summing them.

Continuous predictor variables were categorized based on clinical guidelines and literature. The reference class was determined by the direction of the beta coefficients using the ‘last class’ for negative coefficients and the ‘first class’ for positive ones. A multiplication factor was calculated by subtracting the reference class mean from each class mean. Each class’s beta coefficient was derived by multiplying the continuous variable’s beta by this factor and dividing by the smallest coefficient to assign scores (Table 6).

**Table 6:** Simplified risk score development of maternal near-miss predictors based on beta coefficient in a public health facility, Bahir Dar City, Northwest Ethiopia, 2024.

Prognostic predictors	β	Interval	WM	WM-WMf	β(WM-WMf)	Score
Age in years	0.12	18-24	21	0	0	0
		25-34	29.5	8.5	1.02	3
		35^+^	38	17	2.06	7
Residence-rural	0.68				0.68	2
Decision-making-husband/3^rd^ party	0.74				0.74	3
Pregnancy intention -unintended	0.47				0.47	2
Female genital mutilation-yes	0.42				0.42	1
Systolic blood pressure in mmHg	0.04	< 120	117	0	0	0
		120–129	124.5	7.5	0.30	1
		130–139	134.5	17.5	0.70	2
		≥ 140	145.5	28.5	1.14	4
Time of ANC initiation in weeks	0.15	Within 12	9	0	0	0
		13-16	14.5	5.5	0.83	3
		Above 16	18	9	1.35	5
Middle upper circumference in cm	−0.13	< 23	19.95	−7.05	0.92	3
		≥23	27	0	0	0
Obstetric morbidity history-yes	0.56				0.56	2
C/S history –yes	0.86				0.86	3
Hemoglobin value in g/dl	−0.48	7-8.9	7.95	−5.05	2.42	8
		9-10.9	9.95	−3.05	1.46	5
		≥11	13	0	0	0

The prognostic index model is expressed as: PI = 0.12 × age + 0.68 × residence + 0.74 × decision-making power+0.47 × intention of pregnancy+0.15 × timing of ANC initiation-0.13 × MUAC+0.86 × history of C/S+0.42 × FGM + 0.04 × systolic blood pressure-0.48 × hemoglobin +0.56 × history of obstetric morbidity. For example, a 36 year of age pregnant woman from a rural residence, with a health care decision made by her husband, has a history of female genital mutilation, has a history of C/S, started ANC at 17 weeks, with unintended pregnancy, has a systolic blood pressure of 145 mmHg, MUAC of 20 cm, hemoglobin value of 8 mg/dl and has obstetric history has a risk score of 40.

### Risk classification

**Fig 8:**
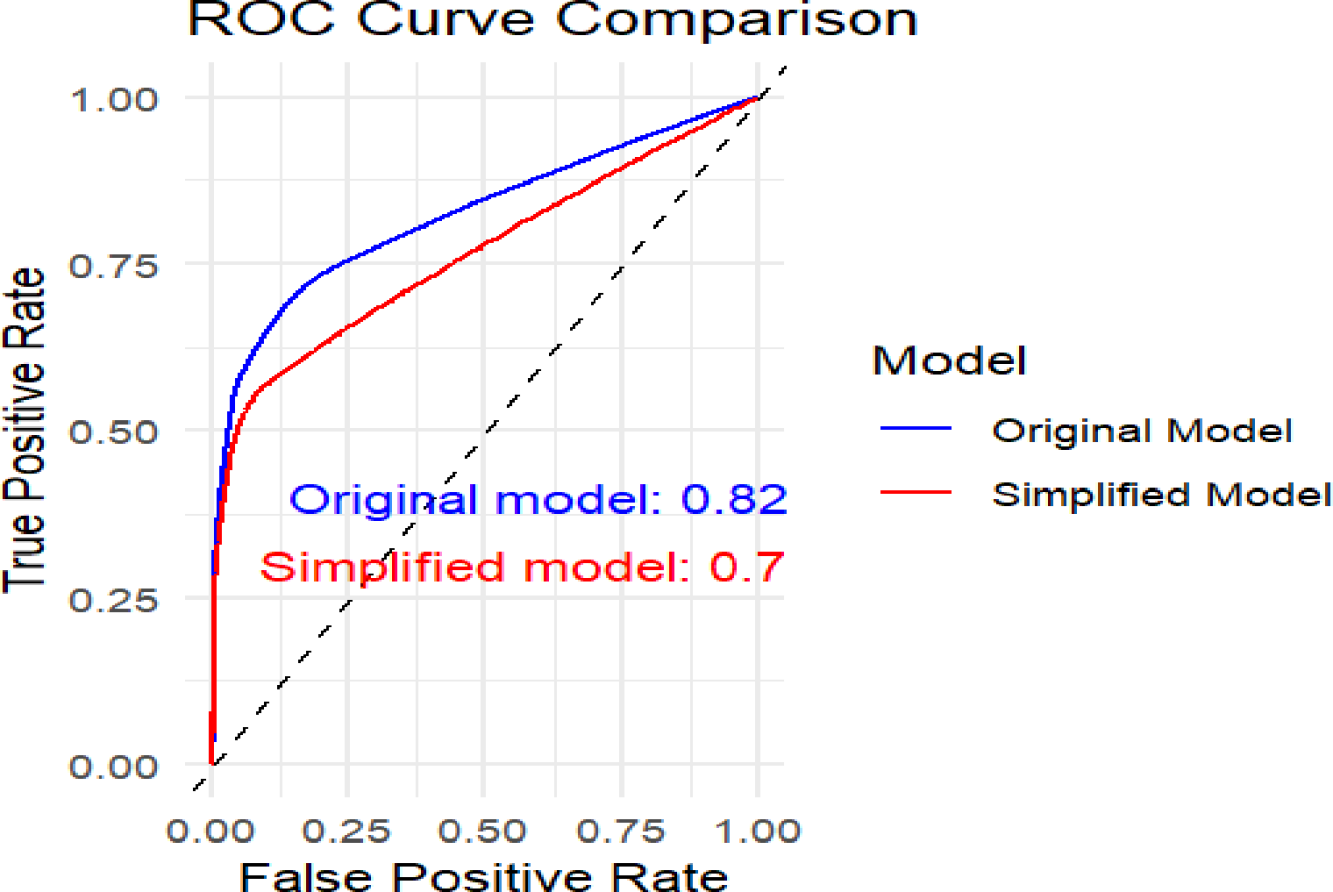
The receiver operating curve to assess discrimination performance for the original and the simplified risk score.

The validated model created a simplified risk score for clinical use. The risk score’s C-index of 70% demonstrated a good capacity for discriminating, almost similar to the original model’s C-index of 82%. This indicates that there was an 86.00% chance that a randomly chosen near-to-death woman would have a higher risk score than a randomly selected patient who survived (Fig 8).

The study’s highest possible overall risk score was 40. Based on the model’s Youden index (13 ideal cut-off points) of 0.01 in the model, the score was categorized into three groups for ease of interpretation in clinical settings. As a result, women with a score of less than 13 were classified as having a low risk of maternal near-miss, those with 13–15 points as medium, and women with 16 points or more as high risk. Of the 161 events, 81(50.31%) and 31(19.25%) were in the high- and medium-risk groups, respectively (Table 7).

**Table 7:** Risk stratification for maternal near-miss using simplified prediction score in a public health facility, Bahir Dar City, Northwest Ethiopia, 2024.

Risk score	Censored	Event	Total (%)
High risk	68(4.52)	81(50.31)	149(8.95)
Low risk	1266(84.23)	45(27.95)	1311(78.79)
Medium risk	169(11.24)	31(19.25)	204(12.26)
Total	1503(100)	161(100)	1664(100)

Maternal near-miss risk score=(age (15–24 year)*0)+(age (25–34 year)*3)+(age (35 + year)*7) + (residence (urban)*0) + (residence (rural)*2)+(decision making power (herself) *0)+(decision making power (third party)*3)+(pregnancy intension (intended)*0)+(pregnancy intension (unintended)*2)+(female genital mutilation (no)*0)+(female genital mutilation (yes)*1)+(systolic blood pressure (<120 mmHg)*0)+(systolic blood pressure (120–129 mmHg)*1)+(systolic blood pressure (130–139 mmHg)*2)+(systolic blood pressure (≥140 mmHg)*4)+(time of antenatal initiation (≤12 week)*0)+(time of antenatal initiation(13–16week)*3)+(time of ANC initiation (≥17week)*5) +(MUAC(<23 cm)*3)+(MUAC (≥23 cm)*0) + (history obstetric morbidity (no)*0)+(history obstetric morbidity (yes)*2)+(history of C/S (no)*0) +(history of C/S (yes)*3)+(hemoglobin(7-8.9g/dl)*8)+(hemoglobin(9–10.9 g/dl)*5) + (hemoglobin (≥11g/dl)*0).

#### Nomogram development.

**Fig 9:**
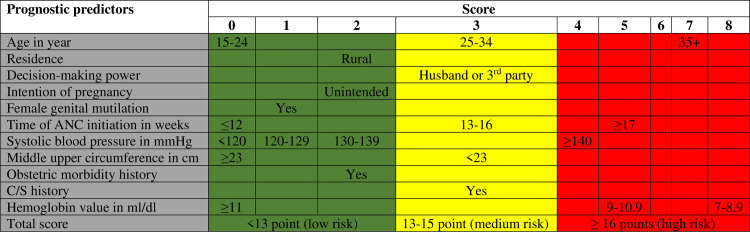
Nomogram for predicting maternal near-miss in a public health facility, Bahir Dar City, northwest Ethiopia, 2024.

A nomogram was created to simplify the process. Individual patient scores can be calculated by measuring the variables on the left and finding the corresponding score at the top. The scores can then be summed to determine the risk classification group (Fig 9).

## Discussion

Maternal near-miss is a key indicator of obstetric care quality and maternal health [38]. It indicates healthcare system effectiveness, identifies service delivery gaps, and promotes quality improvement [2]. Evidence on maternal near-miss helps healthcare providers and policymakers enhance maternal health outcomes [39]. Hence, this prospective follow-up study determined an incidence density rate and developed a prognostic predictive model for maternal near-miss in Bahir Dar City, Northwest Ethiopia.

An incidence density rate of 1.94 per 1,000 woman-weeks was found in the present study. This statistic shows that roughly two women for every 1,000 weeks of observation had serious obstetric complications. This high incidence density rate of maternal near-miss points to serious flaws in the healthcare system, including poor management of pregnancy and delivery problems, and limited access to emergency services. These problems point to the urgent need for legislative changes to guarantee the effectiveness and accessibility of maternity healthcare. Enhancing provider education is also obviously required to give medical personnel the tools they need to identify and address issues as soon as they arise. This circumstance also emphasizes how crucial it is to allocate resources strategically so that hospitals are prepared to manage crises and offer complete maternal care [40]. Studies in different regions may report varying incidence rates, reflecting local healthcare challenges and the effectiveness of maternal health interventions [41]. An increasing maternal near-miss rate demands improved training, better access to care, enhanced management protocols, evaluation of maternal health programs, and effective allocation of resources to reduce morbidity and mortality [42].

The prognostic predictive model of maternal near-miss was developed using maternal age, residence, decision-making power, pregnancy intention, timing of ANC initiation, MUAC, female genital mutilation, history of C/S, systolic blood pressure, hemoglobin, and history of obstetric morbidity. These prognostic features of the model were less costly and easily identifiable, including basic demographic and clinical factors. Easily recognizable and measurable variables are essential for integrating prediction models into clinical practice. Their clarity allows healthcare professionals to incorporate them into daily workflows, facilitating smoother applications and enabling informed decision-making in patient care [21]. They also simplify data collection, which is vital in large-scale studies with limited resources, enabling faster data acquisition that is essential for making timely decisions [43]. Additionally, easily measurable variables promote standardization of the model at different times or settings for broad application of the model [44].

The maternal near-miss model has demonstrated good discrimination, achieving a C-index of 0.82(95% CI: 0.80–0.85). The current model’s lower discrimination ability (AUC 0.82) compared to the Nigerian study (AUC 0.92) [27] can be attributed to several factors. The case-control design in the previous study typically focuses on distinct outcomes, providing clearer associations, while the prospective follow-up in the current study may introduce variability over time. Additionally, the Nigerian study’s smaller sample size may have been more homogenous, enhancing its discriminatory power. The predictor selection in the Nigerian study was optimized through binary logistic regression, which often identifies impactful features more effectively than the current model’s reliance on clinical relevance and literature. Variations in demographics and clinical conditions may also influence performance. Lastly, the current model may experience greater censoring, affecting survival probability estimation and overall discrimination.

The current C-index value indicates that the model has good performance in distinguishing the women who experience maternal near-miss and those who do not., Good discriminatory models successfully distinguish between high- and low-risk patients, allowing for more effective resource allocation and customized treatments to enhance patient care [45]. They support informed clinical decisions, enhance outcomes, reduce unnecessary procedures, and ensure evidence-based practice by optimizing risk identification and validating clinical guidelines [45]. Healthcare providers can utilize this model to identify women at high risk for severe complications, enabling timely interventions and potentially reducing maternal morbidity and mortality.

The model calibration also showed a slope that is closely aligned with 45 degrees. A slope of 45 degrees on a calibration plot indicates that as the predicted probability rises, the actual probability of the event also increases correspondingly [46]. This alignment demonstrates that the model is well-calibrated. Effective calibration is crucial for the practical use of predictive models, as it boosts healthcare providers’ confidence in relying on these predictions for decision-making [46].

Additionally, we used DCA to assess the model’s clinical and public health impact. The net benefit of the model-based treatment decision is greater than that of treating none or all women. Decision curve analysis offers a framework to evaluate whether a prediction model improves patient outcomes compared to standard strategies, emphasizing its clinical value [47]. It calculates net benefits, helping clinicians understand trade-offs between true and false positives while visualizing model impacts. Decision curve analysis also facilitates comparisons among models, enhances guideline transparency, and encourages the development of clinically useful models [48].

The validated model successfully developed a simplified risk score intended for clinical application. This risk score achieved a good ability to discriminate between patients at different risk levels, performing nearly as the same as the original model. Simplified risk score is a crucial metric in evaluating the performance of predictive models, as it reflects the model’s ability to correctly rank the risk of events [49]. The close performance to the original model indicates that the simplification did not significantly compromise the model’s predictive power, allowing for easier implementation in routine clinical practice [46].

## Implications

This study has major clinical implications for clinicians dealing with severe maternal morbidity. By identifying patients who are at high risk for a maternal near-miss, clinicians can intervene early and improve outcomes for severe obstetric complications. Prognostic algorithms can detect changes in the anticipated likelihood of severe maternal morbidity, allowing prompt interventions. Furthermore, the constructed model can help tailor treatment regimens based on patient-specific features. This clinically relevant model also helps educate patients and their families about the expected outcomes of obstetric events and assists researchers in recruiting participants for intervention trials.

## Limitations

The researchers recognize that collecting information on certain factors that happened before the maternal near-miss event could introduce the possibility of recall bias. The application of WHO maternal near-miss screening criteria may underestimate the detection of maternal near-miss. The maternal near-miss was determined only among women who started the ANC service within 20 weeks of GA at the health facility. Hence, the rate of maternal near-miss may be influenced by maternal near-miss at home and started after 20 weeks of GA.

## Conclusion

The incidence density rate of maternal near-miss was high in the present study. A maternal near-miss model was developed using maternal age, residence, decision-making power, pregnancy intention, timing of antenatal care initiation, female genital mutilation, history of cesarean section, systolic blood pressure, hemoglobin, middle upper arm circumference, and history of obstetric morbidity. This model demonstrated good discrimination and calibration ability in distinguishing between survived and non-survived participants. Before applying the model, the researchers recommended that external validation in different settings evaluate the model’s generalizability (S2 File).

## Supporting information

S1 File Data collection tools. (DOCX)

S2 File Minimal dataset. (CSV)
