# Supplementary material for: Maternal near-miss prediction model development in Bahir Dar city administration, Northwest Ethiopia
Source: PLoS One. 2025 Jul 10;20(7):e0328069. doi: 10.1371/journal.pone.0328069 (PMC12244725; doi:10.1371/journal.pone.0328069)
Supplement: S1 File — (DOCX) [file pone.0328069.s001.docx]

1. **Interview Questionnaire**

| No | Variable | | Response | Skip |
| --- | --- | --- | --- | --- |
|  |  |  |  |  |
| **Part I: Socio-demographic characteristics** | | | | |
| 101 | Age | | ……………………… year |  |
| 102 | Religion | | Orthodox…………..…….…….1  Muslim………………….…….2  Protestant……………………...3  Catholic………………...……..4  Other specify…………….……5 |  |
| 103 | Ethnicity | | …………….. |  |
| 104 | Educational status of woman | | Cannot read and write…………1  Can read and write…………….2  Primary (grade 1-8) ………...…3  Secondary (grade 9-12) ……….4  College and above…….…….…5 |  |
| 105 | Occupation of woman | | Governmental employee………1  Private employee ……...………2  Merchant………………………3  Farmer……………………...…4  House wife………...……...…...5  Other(specify)………...……….6 |  |
| 106 | Marital status of woman | | Married…………………….….1  Single………………………….2  Divorced……………...……….3  Widowed…………………...…4  Other specify………………….5 | 109 |
| 107 | Occupation of husband | | Governmental employee.…...…1  Private employee ...….…..……2  Merchant………………………3  Farmer…………………….......4  Other(specify)…………...…….5 |  |
| 108 | Educational status of husband | | Cannot read and write...………1  Can read and write…………….2  Primary (grade 1-8) ……..……3  Secondary (grade 9-12) …...….4  College and above…….…....…5 |  |
| 109 | Residence | | Urban…………….……………0  Rural………………...….….….1 |  |
| 110 | Who is responsible for decision-making? | | Respondent herself…………....1  Respondent& her husband / partner………………………...2  Husband/partner alone………...3  Others, specify………………...4 |  |
| **Part II: Wealth index** | | | | |
| 201 | Ownership of the house | | Own…………………………1  Rent…………………………2 |  |
| 202 | Main source of drinking water? | | Piped into dwelling…………1  Public tap/ standpipe……......2  Protected dug well……….…3  Unprotected dug well…….…4  Protected spring…………….5  Unprotected spring………….6  Other, specify……………….7 |  |
| 203 | Where is the water source located? | | In own dwelling…………......1  In own yard/plot…………….2  Elsewhere……………...……3 |  |
| 204 | How long does it take to go there, get water, and come back? | | ……………………... minutes  Don’t know |  |
| 205 | Is there toilet facility? | | No………………………….0  Yes…………………………1 | 207 |
| 206 | If yes, what type of latrine? | | Ventilated pit latrine…….….1  Pour and flush………........…2  Other, specify……………….3 |  |
| 207 | What type of fuel does the household use for cooking? | | Electricity……………………1  Natural gas…………………...2  Biogas………………………..3  Kerosene…………..…………4  Charcoal………………………5  Wood………………………….6  Straw/shrubs/grass…………….7  Agricultural crop………...……8  Animal dung…………………..9  Other specify…………………. |  |
| Are the following materials available in the household? | | | | |
| 208 | Electricity | | No……………………...……..0  Yes…………………….……...1 |  |
| 209 | Radio | | No……………………...……..0  Yes…………………….……...1 |  |
| 210 | Television | | No……………………...……..0  Yes…………………….……...1 |  |
| 211 | Fixed telephone | | No……………………...……..0  Yes…………………….……...1 |  |
| 212 | Computer | | No……………………...……..0  Yes…………………….……...1 |  |
| 213 | Refrigerator | | No……………………...……..0  Yes…………………….……...1 |  |
| 214 | Table | | No……………………...……..0  Yes…………………….……...1 |  |
| 215 | Chair | | No……………………...……..0  Yes…………………….……...1 |  |
| 216 | Bed with cotton/ sponge mattress | | No……………………...……..0  Yes…………………….……...1 |  |
| 217 | Electric mitad | | No……………………...……..0  Yes…………………….……...1 |  |
| 218 | Kerosene lamp/ preassure lamp | | No……………………...……..0  Yes…………………….……...1 |  |
| Does any member of this household own: | | | | |
| 219 | Watch | | No……………………...……..0  Yes…………………….……...1 |  |
| 220 | Mobile phone | | No……………………...……..0  Yes…………………….……...1 |  |
| 221 | Bicycle | | No……………………...……..0  Yes…………………….……...1 |  |
| 222 | Motorcycle | | No……………………...……..0  Yes…………………….……...1 |  |
| 223 | Animal drawn cart | | No……………………...……..0  Yes…………………….……...1 |  |
| 224 | Cart or truck | | No……………………...……..0  Yes…………………….……...1 |  |
| 225 | Bajaj | | No……………………...……..0  Yes…………………….……...1 |  |
| 226 | Saving account | | No……………………......…...0  Yes…………………….……...1 |  |
| 228 | Car | | No……………………...……..0  Yes…………………….……...1 |  |
| 229 | House’s main floor material? | | Natural (soil)……............…….1  Mud .……………............…….2  Concrete……………..…..……3  Ceramics…………………...….4  Other, specify……………...….5 |  |
| 230 | House’s main roof material? | | Grass/mud roof…………….….1  Corrugated iron sheet……...….2  Other, specify…………...….…3 |  |
| 231 | Household main wall material? | | Wood with Mud……………....1  Cane/palm/trunks/reed………..2  Wood with mud………………3  Concrete………………………4  Ceramic……………………….5  Other, specify…………...…….6 |  |
| 232 | Number of members sleep per bed room | | ……………………...in number |  |
| Does the household have the following domestic animals? | | | | |
| 233 | Cows/bulls | | No……………………...…...…0  Yes………………………….…1 |  |
| 234 | Other cattle | |  |  |
| 235 | Horse/donkey/mules | | No……………………...…...…0  Yes………………………….…1 |  |
| 236 | Goats | | No……………………...…...…0  Yes………………………….…1 |  |
| 237 | Sheep | | No……………………...…...…0  Yes………………………….…1 |  |
| 238 | Chickens and other poultry | | No……………………...…...…0  Yes………………………….…1 |  |
| 239 | Beehives/ bees | | No……………………...…...…0  Yes………………………….…1 |  |
| 240 | Do you have your own agricultural land? | | No……………………...…...…0  Yes………………………….…1 | 301 |
| 241 | How many hectares (timade) of agricultural land? | | …………….in hectare (timade) |  |
| **Part III: Clinical related factors** | | | | |
| 301 | Last normal menstrual period | ……/……../……..date/month/year | |  |
| 302 | When you start the first ANC contact in the current pregnancy? | | ………………..………....in year |  |
| 303 | What was your desire in the current pregnancy? | | Planned………………………..1  Unplanned but wanted………...2  Unplanned unwanted……...…..3 | 305 |
| 304 | Why unplanned? | | …………………… |  |
| 305 | Did you have a history of pregnancy? | | No……………………...…...…0  Yes……………….………....…1 | 318 |
| 306 | What are the number of fetuses in a previous pregnancy? | | Single…………...….……....….0  Multiple……………...…….….1 |  |
| 307 | How many times did you get pregnant? | | ….....................................number |  |
| 308 | How many times did you deliver? | | ….....................................number |  |
| 310 | Did you have a history of stillbirth ever? | | No……………………...…...…0  Yes……………….………....…1 | 312 |
| 311 | Number of stillbirths ever | | ……………...………number |  |
| 312 | Did you have a history of abortion ever? | | No……………………...…...…0  Yes……………….………....…1 | 313 |
| 313 | Number of abortions ever | | …………………………number |  |
| 314 | After delivery of the index child, how many months you took to get pregnant? | | …………………….........months |  |
| 315 | What was the mode of delivery in the last index pregnancy? | | Vaginal…………….….1  C/S………….…….…...2  Assisted instrumental….3 |  |
| 316 | Did you have a history of obstetric morbidities in the index pregnancy? | | No……………………...…...…0  Yes……………….………....…1 |  |
| 317 | What were these obstetric morbidities? (Multiple response is possible) | | Hypertension……………..…...1  Antepartum hemorrhage……...2  Postpartum hemorrhage………3  Obstructed labor………………4  Prolonged labor ……........……5  Other specify………………….6 |  |
| 318 | Did genital mutilation performed on you? | | No……………………...……...0  Yes…………………………….1 |  |
| 319 | Did you have history of medical morbidities? | | No……………………...…...…0  Yes……………….………....…1 | 321 |
| 320 | What were this medical morbidity? (Multiple response is possible) | | Anemia……………………..…1  Hepatitis…………………...….2 Tuberculosis…………...…...…3  DM……………………………4  Hypertension………………….5  HIV/AIDS…………………….6  Heart diseases……...………….7  Other specify……………….…8 |  |
| 321 | Do you have medical morbidity in current pregnancy? | | No……………………...…...…0  Yes……………….………....…1 | 401 |
| 322 | What are these medical morbidities? (Multiple response is possible) | | Anemia……………………..…1  Hepatitis…………………...….2 Tuberculosis…………...…...…3  DM……………………………4  Hypertension………………….5  HIV/AIDS…………………….6  Heart diseases……...………….7  Other specify……………….… |  |
| **Part IV: Accessibility related factors** | | | | |
| 401 | Is there accessibility of road towards to health facility? | | No…………………………….0  Yes……………………………1 |  |
| 402 | Are there accessibility of transportation towards the nearest health facility? | | No…………………………….0  Yes……………………………1 |  |
| 403 | What is the distance to the nearest health facility? | | …………….…………...….km |  |

1. **Measurement**

| No | **Variable** | **Response** | **Skip** |
| --- | --- | --- | --- |
|  |  |  |  |
| **Part I: Anthropometry measurements** | | | |
| 101 | Weight | ……………………………. kg |  |
| 102 | Height | ……………………… centi-meter |  |
| 103 | Middle upper arm circumference | ……………………….centi-meter |  |
| **Part II: Vital sign measurements** | | | |
| 201 | Systolic blood pressure | ………………………..…… mHg |  |
| 202 | Diastolic blood pressure | ………………………………mHg |  |

1. **Extraction checklists**

| No | | Variable | | | Response | | Skip |
| --- | --- | --- | --- | --- | --- | --- | --- |
|  |  |  |  |  |  |  |  |
| **Part I: Obstetric Morbidity** | | | | | | | |
| 101 | | Does an obstetric problem exists in the current pregnancy? | | | No…………….……0  Yes…………………1 | | 121 |
| 102 | | What were the occurred obstetric morbidities? (Multiple answers is possible) | | | Preeclampsia…….…1  Eclampsia………….2  Anemia.……………3  Antepartum hemorrhage…......…4  Postpartum hemorrhage…….….5  Prolonged………..…6  PROM……………...7  Obstructed labor……8  Other specify…...….9 | |  |
| 103 | | WHO Clinical based criteria modified for Sub-Saharan Africa | Acute cyanosis | | No……….…………0  Yes…………………1 | |  |
| 104 | |  | Gasping | | No……….…………0  Yes…………………1 | |  |
| 105 | |  | Respiratory rate> 40or< 6/min | | ………. breath/minute | |  |
| 106 | |  | Shock | | No……….…………0  Yes…………………1 | |  |
| 107 | |  | Oliguria non responsive to fluids or diuretics | | No……….…………0  Yes…………………1 | |  |
| 108 | |  | Failure to form clots | | No……….…………0  Yes…………………1 | |  |
| 109 | |  | Loss of consciousness lasting 12h | | No……….…………0  Yes…………………1 | |  |
| 110 | |  | Cardiac arrest | | No……….…………0  Yes…………………1 | |  |
| 111 | |  | Stroke | | No……….…………0  Yes…………………1 | |  |
| 112 | |  | Uncontrollable fit/total paralysis | | No……….…………0  Yes…………………1 | |  |
| 113 | |  | Jaundice in the presence of pre-eclampsia | | No……….…………0  Yes…………………1 | |  |
| 114 | |  | Eclampsia | | No……….…………0  Yes…………………1 | |  |
| 115 | |  | Uterine rupture | | No……….…………0  Yes…………………1 | |  |
| 116 | |  | Pulmonary edema | | No……….…………0  Yes…………………1 | |  |
| 117 | |  | Severe malaria | | No……….…………0  Yes…………………1 | |  |
| 118 | |  | Severe pre-eclampsia with ICU admission | | No……….…………0  Yes…………………1 | |  |
| 119 | |  | Sepsis or severe systemic infection | | No……….…………0  Yes…………………1 | |  |
| 120 | |  | Severe abortion complications | | No……….…………0  Yes…………………1 | |  |
| 121 | | End of follow-up time | | | ........../……/….day/month/year | |  |
| 122 | | What was the reason for the loss to follow up? | | | Residence change ......1  Refusal ………….......2  Death……………..…3  Other specify …………4 | |  |
| Part 2: lab investigations | | | | | |  | |
| 201 | Hemoglobin……………….. | | | …………………………..……gm/dl | |  | |
| 202 | Hematocrit..…………….. | | | …………………………………….% | |  | |
